# On-chip single photon filtering and multiplexing in hybrid quantum photonic circuits

**DOI:** 10.1038/s41467-017-00486-8

**Published:** 2017-08-30

**Authors:** Ali W. Elshaari, Iman Esmaeil Zadeh, Andreas Fognini, Michael E. Reimer, Dan Dalacu, Philip J. Poole, Val Zwiller, Klaus D. Jöns

**Affiliations:** 1 Quantum Nano Photonics Group, Department of Applied Physics, Royal Institute of Technology (KTH), 106 91 Stockholm, Sweden; 2 Kavli Institute of Nanoscience Delft, Delft University of Technology, 2628 CJ Delft, The Netherlands; 3 Single Quantum, 2628 CJ Delft, The Netherlands; 4 Institute for Quantum Computing and Department of Electrical & Computer Engineering, University of Waterloo, Waterloo, Ontario Canada N2L 3G1; 5 National Research Council of Canada, Ottawa, Ontario Canada K1A 0R6

## Abstract

Quantum light plays a pivotal role in modern science and future photonic applications. Since the advent of integrated quantum nanophotonics different material platforms based on III–V nanostructures-, colour centers-, and nonlinear waveguides as on-chip light sources have been investigated. Each platform has unique advantages and limitations; however, all implementations face major challenges with filtering of individual quantum states, scalable integration, deterministic multiplexing of selected quantum emitters, and on-chip excitation suppression. Here we overcome all of these challenges with a hybrid and scalable approach, where single III–V quantum emitters are positioned and deterministically integrated in a complementary metal–oxide–semiconductor-compatible photonic circuit. We demonstrate reconfigurable on-chip single-photon filtering and wavelength division multiplexing with a foot print one million times smaller than similar table-top approaches, while offering excitation suppression of more than 95 dB and efficient routing of single photons over a bandwidth of 40 nm. Our work marks an important step to harvest quantum optical technologies’ full potential.

## Abstract (web summary)

Combining different integration platforms on the same chip is currently one of the main challenges for quantum technologies. Here, Elshaari et al. show III-V Quantum Dots embedded in nanowires operating in a CMOS compatible circuit, with controlled on-chip filtering and tunable routing.

## Introduction

The implementation of the linear optics quantum computation scheme^1^ requires large resources and remarkable photon detection and generation efficiencies^2^. Thus, the progress of quantum information processing^3^ and sensing implementations^4^ using quantum states of light strongly depends on miniaturization and simultaneous integration of the main elements of a quantum circuit, i.e., sources, optical gates, and detectors^5^. Photonic integration provides means of miniaturization to efficiently integrate elements of the quantum circuit on-chip with low insertion losses, high efficiencies^6, 7^, and high density. Generally, the type of application determines which photonic platform is used, but there are some universal requirements in a quantum photonic circuit regardless. These requirements are as follows: deterministic integration of multiple on-demand selected single-photon sources^7, 8^; demonstration of complex photonic circuits for qubit manipulation^9–11^; and on-chip detection^6^. The key ingredient to realize such a complete system is on-chip spectral filtering and multiplexing of multiple quantum emitters^12^. Several reports addressed single-photon filtering and on-chip excitation suppression^9, 10, 13^, but were limited to quantum emitters of probabilistic nature in nonlinear optical waveguides. In contrast, semiconductor quantum dot (QD) single-photon sources^14^ are suitable for on-demand photon generation^15^, but there are no reports of on-chip filtering and multiplexing of QD single-photon sources. QD-based single-photon sources have an additional advantage that they also offer the possibility for on-chip electrical excitation^16^ and wavelength tunable entangled photon emission^17, 18^. In conjunction with two-photon interference visibilities approaching unity^19^ and high photon flux rates^20, 21^, QDs are therefore attractive for on-chip quantum optical applications. However, there are challenges to realize III–V quantum photonic circuits^22–24^. These challenges include deterministic integration of selected QDs into optical waveguides/cavities, efficient filtering of specific quantum transitions within the emission spectrum, on-chip pump suppression, and multiplexing of multiple QDs.

In our work, we overcome all of the discussed challenges by realizing hybrid integrated quantum photonic circuits. Our circuits generate quantum light on-chip relying on a fully integrated and tunable spectral filtering to isolate optically active QD transitions. Moreover, to emphasize the controlled nature of the process and the vast range of new applications it enables, we demonstrate on-chip wavelength division multiplexing of quantum emitters with tunable routing. Finally, we present an in-plane pumping scheme and realize a multi-quantum emitter circuit with independently selected and deterministically integrated quantum emitters.

## Results

### Hybrid integration

**Fig. 1:**
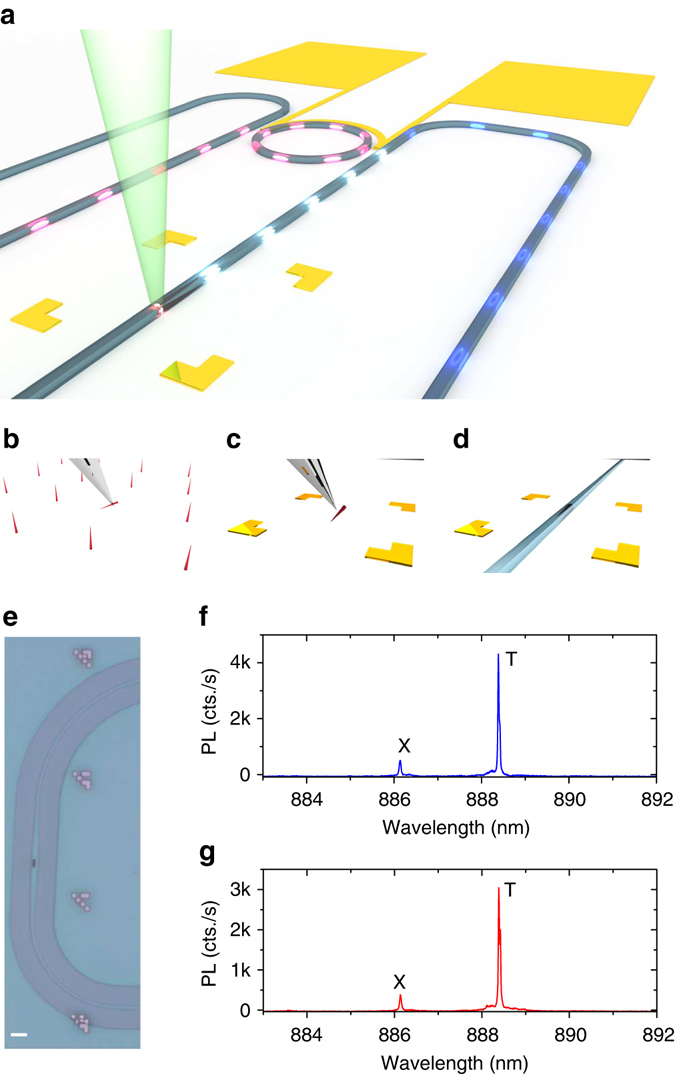
Hybrid quantum photonic circuit. **a** Schematic view of the fabricated hybrid quantum photonic circuit, consisting of an InAsP QD in an InP nanowire (*ruby* coloured) that is integrated with a SiN waveguide (*blue*) and on-chip tunable ring resonator filter. The ring resonator filter is tuned by applying voltage to the gold contacts (*orange*). The out-of-plane laser (*green*) excites the QD, which emits single photons (*ellipsoids*) into the waveguide. **b**–**d** The CMOS compatible process flow for integrating the nanowire-based quantum light sources within the hybrid quantum photonic circuit. Using a custom-built tungsten nanomanipulation tool, the nanowires are transferred from the growth chip to the selected photonic circuit substrate. **e** A microscope image of an integrated single-photon source with a SiN photonic waveguide, the *scale bar* has length of 5 µm. Emitted photons are coupled to the SiN photonic channel with the possibility to collect both forward and backward photons independently. The photonic circuits are fabricated with respect to the transferred quantum emitters as described in the Methods section. **f**, **g** The collected forward and backward photoluminescence (PL) from the nanowire QD, respectively. T and X represent the trion and exciton emission lines, respectively

Figure 1a shows an artistic view of the hybrid quantum photonic circuit developed in our work, comprising of a nanowire-based quantum light source embedded in a photonic waveguide with tunable ring resonator filter. The quantum emitter consists of an InAsP QD embedded in a pure wurtzite InP nanowire. Details on the nanowire growth can be found in ref. ^25^. Emission properties such as broadening and indistinguishability of these quantum emitters have been characterized extensively^26, 27^. After pre-characterizing the nanowire QDs on the growth chip, we select emitters based on their emission wavelength, brightness, and line width, and then transfer them to the desired location using a custom-built nanomanipulation tool^7^ (see Methods section and Supplementary Note 7 for more information). Next, the photonic channel is carefully designed to encapsulate the transferred nanowire-based quantum emitter. The photonic channel consists of a 200 nm-thick silicon nitride (SiN) layer processed into an 800 nm-wide waveguide and cladded with a polymethyl methacrylate (PMMA) layer. Figure 1b–d summarizes the quantum photonic circuit fabrication process where we first deterministically position the pre-selected nanowire-based quantum emitter and then construct the photonic waveguide around it. A detailed description of the fabrication steps and nanowire nanomanipulation can be found in the Methods section. Figure 1e shows a microscope image of a nanowire-based quantum emitter integrated in a photonic waveguide. The nanowire is encapsulated in the SiN waveguide, allowing for ease of excitation and collection in the forward and backward directions with a combined coupling efficiency of ~ 24%^7^. Figure 1f, g shows the collected photoluminescence (PL) in the forward and backward directions of the photonic channel, respectively. If desired, this configuration can directly act as an on-chip non-polarizing beamsplitter, and the observed ¾ ratio between the forward and backward directions can be engineered at will. The ratio mainly depends on the nanowire shape^7^ and differences in the out-of-chip coupling and propagation losses of the two waveguides (see Methods).

### On-chip tunable filtering

**Fig. 2:**
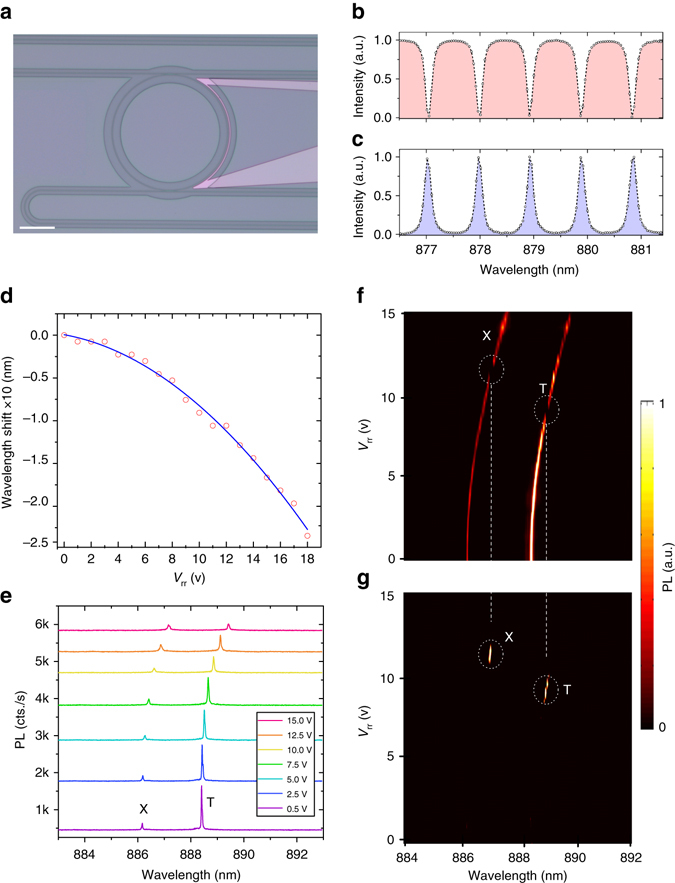
On-chip single-photon routing. **a** A microscope image of the ring resonator filter. The filter transmission is controlled with a titanium resistor, the *scale bar* has length of 70 µm. Details of the fabrication process are provided in the Methods section. Graphs **b** and **c** show the through-port and drop-port transmission of the ring resonator, respectively. **d** Tuning of the ring resonator filter as a function of the filter voltage (*V*_rr_).The *red circles* show the measured shifts while the *blue line* is a fit. The resonances are blue shifted by design, which is achieved by means of the large negative thermo-optic coefficient of the PMMA top cladding. **e** Tuning of the QD transition as a function of the ring resonator voltage. In contrast to the observed shift for the ring resonator, the QD emission red shifts for increasing voltage as expected due to the increase in temperature. Graphs **f** and **g** show the results of selectively routing a unique transition of the QD between the drop-port and through-port of the ring resonator. As we tune the filter resonance using the integrated heater, a single emission line can be tuned in and out of resonance, thus routing single photons between the through-port and drop-port

As shown in Fig. 2a, the emitted photons from the nanowire QD in the forward direction are routed to an electrically tunable 70 µm-radius ring resonator. The SiN waveguide width, height, and 180 nm separation between the ring resonator and the bus waveguides are designed to achieve critical coupling for transverse electric (TE) modes (see Supplementary Note 1 for transverse magnetic transmission). Figure 2b, c presents the measured through-port and drop-port transmission of the ring resonator TE mode (electric field parallel to the substrate), respectively. The free spectral range of the resonator is ~ 0.96 nm at the QD emission wavelength with a resonance full width at half maximum of 0.13 nm. To efficiently tune the ring resonator to the QD emission, the top cladding was carefully chosen to have a negative thermo-optic coefficient, ~ 10 times larger than the positive thermo-optic coefficient of the SiN waveguide core^28, 29^. Figure 2d shows the ring resonances shift to shorter wavelength (higher energies) with increasing voltage. In contrast, as shown in Fig. 2e, the QD emission shifts in the opposite direction to longer wavelengths (lower energies) for increasing voltage. This counteracting tuning mechanism of the resonator and the QD enhances the tuning rate. To minimize the thermal coupling between the nanowire and the tunable filter, the PMMA cladding was removed from most of the substrate except for small areas surrounding the photonic channels to preserve optical mode confinement. The voltage range between 0 and 15 V covers already 120% of the total free spectral range of the resonator, we therefore can efficiently select any spectral line in a range of 40 nm without degradation in the performance of the ring resonator. We independently studied the QD emission as a function of chip temperature to estimate the actual temperature of the QD when a certain voltage is applied to the ring resonator filter, see Supplementary Note 2 for measurement details.

As shown in Fig. 2f, g, specific resonant modes of the resonator can be tuned to either the trion (T) or exciton (X) transition of the QD. Thus, single photons are routed from the through-port to the drop-port with more than 15 dB selectivity measured at the through-port. Careful engineering of the resonator allows for selective filtering based on wavelength and/or polarization. This capability becomes important in entanglement type experiments using the QD’s biexciton–exciton cascade. Finally, the versatile nature of SiN photonics allows for a vast range of more complex multi-staged circuitry^30^, with third-order nonlinearity that can be employed to perform on-chip inter- and intra-band single-photon wavelength conversion^31^.

### Demonstration of quantum light on-chip

**Fig. 3:**
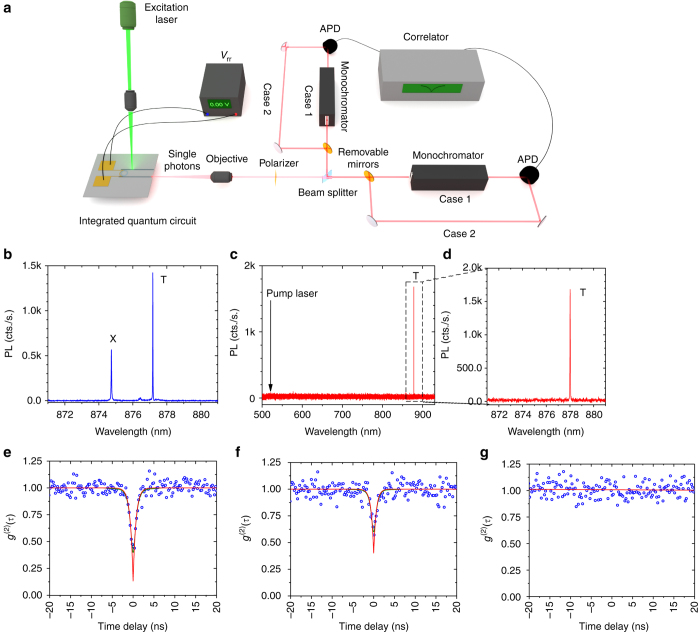
On-chip filtering. **a** Experimental setup showing the hybrid integrated quantum circuit with electrical access to control the integrated filters. The setup allows for both in-plane (via the waveguide) and out-of-plane laser excitation. Details of out-of-chip coupling are included in the Methods section. The collected emission from the waveguide is either coupled to the APDs after filtering with a monochromator (case 1), or it can be directly coupled to the APDs with no external wavelength filtering (case 2). **b** Collected QD emission from the facet of the SiN waveguide in the forward direction. **c** By tuning the on-chip filter, a single QD transition is routed to the drop-port. **d** A close-up of the filtered trion (T) emission line. The QD emission wavelength is slightly different in **b** and **c** or **d** due to different biases applied to the ring resonator filter. Despite the presence of an intense laser for excitation and InP nanowire emission, the filtered spectrum shows only a single QD transition over a broad wavelength range (500–950 nm). **e** Second-order correlation measurement of the QD trion line using an off-chip commercial monochromator for filtering, resulting in a multi-photon probability of *g* ^(2)^(0) = 0.13 ± 0.04 when taking into account the finite temporal resolution of the APDs. **f** Second-order correlation measurement of the QD trion line at the drop-port of the ring resonator after directly coupling it to the APDs. A single-stage ring filter is capable of delivering single photons on-chip with multi-photon probability *g* ^(2)^(0) = 0.41 ± 0.05. The results show the excellent performance of the integrated ring resonator filter as compared to the bulky off-chip monochromator. **g** Second-order correlation measurement without any on-chip and off-chip filtering. The results show the expected Poissonian statistics of coherent (uncorrelated) emission. In **e**, **f**, the *blue circles* show the raw data, the *green line* represents a fit, and the *red line* represents the fit considering the finite detector response (see Supplementary Note 4 for more details)

Next, we generate quantum light on-chip without the use of any bulky or lossy external wavelength filters. The experimental setup is shown in Fig. 3a. The QD is excited from the top using a continuous wave 532 nm laser (see Methods for more information about QD excitation and photon collection). The collected emission from the waveguide facet is either filtered through a monochromator, then detected by an avalanche photodiode (APD; case 1), or it can be directly detected by the APDs (case 2). Figure 3b shows the unfiltered emission spectra of the encapsulated nanowire QD. Both the exciton and trion transitions are visible. Figure 3c presents a high-resolution measurement performed on the drop-port emission of the ring resonator over a wide wavelength range (from 500 to 950 nm) after the filter voltage is tuned to align the ring resonator resonance to the QD trion transition. Figure 3d focuses on the filtered trion transition, showing the full suppression of the exciton line. Despite the presence of an intense laser excitation of power density ~ 0.5 µW/µm^2^ from the top, in addition to the exciton and bulk InP nanowire emission, only the selected trion emission line is collected. With our filtering, we achieve an on-chip excitation suppression of more than 95 dB (see Supplementary Note 3 for detailed information about excellent on-chip excitation suppression).

In order to characterize the on-chip ring resonator filter we benchmark its performance against a commercial table-top monochromator, an Acton 750 mm with 1800 grooves per millimeter grating. The bandwidth of the monochromator is ~ 0.02 nm with an overall transmission of ~ 25% and peak attenuation in the rejection band of more than 80 dB. We perform second-order correlation measurements on the trion line in both cases: case 1—using the monochromator as a filter and case 2—bypassing the monochromator and using the on-chip ring resonator filter. Case 1 is shown in Fig. 3e where the multi-photon probability at zero-time delay is *g* ^(2)^(0) = 0.13 ± 0.04 (see Supplementary Note 4 for measurement details and cross-correlation measurements between the trion and the exciton lines). The result for case 2 with only on-chip filtering is shown in Fig. 3f. In the latter case, the multi-photon probability at zero-time delay is *g* ^(2)^(0) = 0.40 ± 0.05, demonstrating the emission from a single quantum emitter on-chip. As a final check, the measurement is repeated without the use of any filtering, shown in Fig. 3g. As expected, without the monochromator or the ring resonator filtering we observe no signature of non-classical light emission. Comparing the previous measurements clearly shows the functionality of our on-chip single-photon filtering using a single-stage ring resonator. The unwanted coupled light to the drop-port can be further reduced by coupled resonators with larger free spectral range or narrower bandwidth^30^.

### Wavelength division multiplexing of quantum sources

**Fig. 4:**
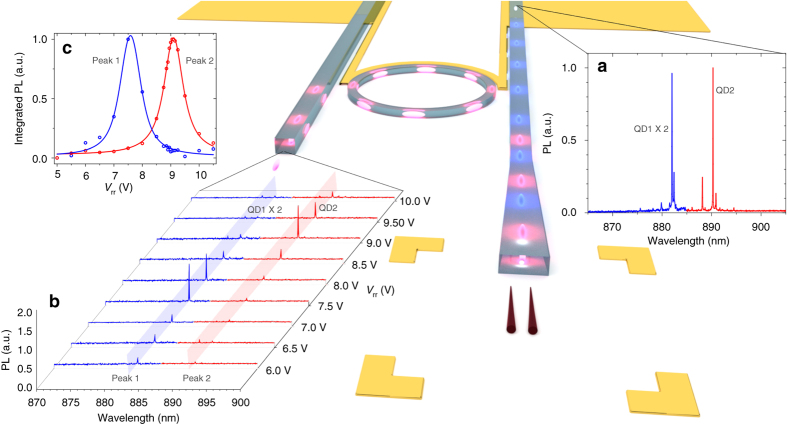
Wavelength mulitplexing of quantum emitters. Artistic image of multiplexing/demultiplexing of two quantum emitters coupled to a photonic circuit with integrated tunable filters. The two nanowires are butt-coupled to a SiN waveguide, each of them emitting photons independently with different colours (depicted as *red* and *blue* in this case). The flexibility of the process allows for the possibility of wavelength and modal multiplexing of selected single-photon sources to an already fabricated and characterized photonic circuit, thus making the process highly deterministic. Details of the fabrication and nanowire transfer process are included in the Methods section. **a** Collected emission from the through-port waveguide, consisting of a wavelength-multiplexed signal from QD1 and QD2. The spectra are highlighted in *red* and *blue* to indicate the individual emission from QD1 and QD2. **b** Selected excitonic transitions of QD1 and QD2 are filtered deterministically to the drop-port waveguide as a function of the on-chip filter tuning voltage. **c** Integrated intensity of QD1 and QD2 in the drop-port as a function of voltage. As the voltage is controlled, QD1 and QD2 follow the Lorentzian shape of the ring resonator transmission function

Having demonstrated the main building blocks of our hybrid quantum photonic circuit, namely quantum emitter integration and filtering of individual exciton transitions, we move to more complex architectures. To increase the on-chip qubit data rate, advanced multiplexing techniques can be adopted from classical fiber-optic communication systems like wavelength-division multiplexing (WDM). However, up to now the missing on-chip filtering hindered the realization of WDM of on-demand quantum sources. Using our tunable ring resonator filter we are able to perform this task. Figure 4 shows a schematic view of a fabricated 2-emitter on-chip WDM channel. For this device the nanowires were butt-coupled to the photonic waveguide (see Supplementary Note 5 for simulation of nanowire-waveguide coupling). Details of the device fabrication and nanomanipulation of the individual nanowires are included in the Methods section. The two QDs can be excited independently or simultaneously to create a quantum multi-wavelength-integrated channel. Afterwards the photons will be selectively decoupled from the main transmission waveguide based on their wavelength. Figure 4a shows the collected emission spectrum from the through-port waveguide. The spectrum consists of a wavelength-multiplexed packet carrying the emission of two QDs, labeled QD1 and QD2, separated by ~ 10 nm. Demultiplexing the emission is performed using the on-chip tunable ring resonator filter, which only couples the resonant optical modes to the drop-port of the photonic circuit. As shown in Fig. 4b, specific transitions of QD1 and QD2 are routed deterministically to the drop-port waveguide as a function of the on-chip filter tuning voltage. In Fig. 4c we present the integrated intensities of QD1 and QD2 in the drop-port as a function of voltage. As we tune the ring resonator, the intensity of QD1 and QD2 follow a Lorentzian shape given by the transmission function of the ring resonator filter. As we increase the voltage further, the QD1 peak PL is decoupled from the drop-port and we couple the emission of the QD2 peak. See Supplementary Note 6 for more WDM measurements. Our device can be extended to incorporate more emitters and pulsed excitation to realize temporal and WDM quantum links on-chip^32^. With the recent advances of generating entangled photon pairs from similar nanowire QDs^33, 34^, it is possible to multiplex/demultiplex entangled photon pairs in complex network architectures.

### In-plane excitation for scalable integration

**Fig. 5:**
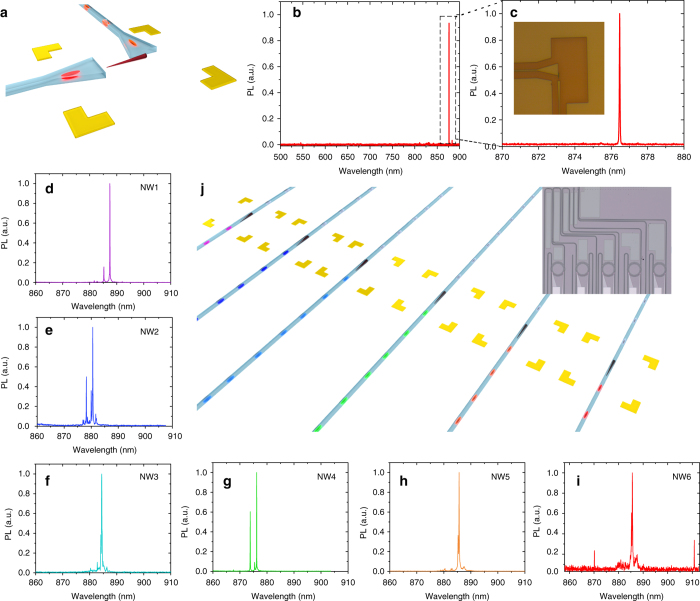
In-plane excitation and large-scale integration. **a** Artistic image of in-plane excitation of the nanowire quantum dot. The nanowire QD emission is collected in the waveguide orthogonal to the pump waveguide. **b** Collected spectrum from the waveguide coupled to the nanowire. **c** A close-up view of the emission spectrum of the QD and a microscope image of the measured device. The spectrum mainly shows a single exciton peak from the QD emission with no measurable signal from the pump (at ~ 632 nm). Pump signal is efficiently suppressed due to the device geometry decoupling the pump photons from the nanowire emission. **d**–**i** The emission spectrum of six independently and deterministically integrated nanowire quantum emitters operating on the same photonic chip. In-plane excitation was used to excite the nanowire quantum dots. This paves the way for large-scale integration and excitation of multiple quantum emitters on-chip either using a single off-chip source or an integrated electrically pumped source with phase shifters and routers^37^. **j** An artistic image of the fabricated device (shown in *inset*) containing six nanowires encapsulated in SiN waveguides

Next, we turn our attention to addressing the scalability of the approach and providing means for large-scale integration. The pumping scheme that was used in the previous measurements relied on out-of-plane excitation, this limits the number of accessible sources and imposes challenges to scale the process as the number of integrated quantum emitters is increased. In contrast, in-plane excitation provides the means to access large number of integrated quantum emitters with a single pump using a set of routers and filters to selectively excite individual QDs. Another advantage is the possibility to engineer the geometry and spectral properties of the photonic structures to realize pump isolation and filtering. An artistic image of our fabricated device is shown in Fig. 5a. The device consists of a nanowire butt-coupled to a SiN waveguide. The base of the nanowire, which contains the quantum emitter, is optically addressed with another waveguide orthogonal to the first one for optical pumping. The optical pumping is performed using a HeNe laser coupled to the SiN waveguide then routed to excite the nanowire QD. The nanowire emission is collected from the orthogonal waveguide then routed off-chip and sent to the spectrometer, the results of the measurement are shown in Fig. 5b. Figure 5c presents a close-up of the nanowire emission with an inset showing a microscope image of the fabricated device containing the nanowire. As shown in the figures, there was no measurable signal from the pump laser at ~ 632 nm despite the fact that there was no on-chip or off-chip filtering used. The spectra mainly consist of a single excitonic line from the QD in addition to bulk wire emission at ~ 825 nm. The pump photons are ultra-efficiently decoupled from the nanowire QD emission due to the orthogonal excitation and collection scheme. This geometry is potentially attractive for performing resonant excitation^15^ of quantum emitters with no additional filtering needed to eliminate the pump photons.

Finally, we would like to emphasize the feasibility for realizing more complex multi-emitter circuits even further. Our current technology allows for transferring wires to selected positions with an average time of 6 min and a success rate of more than 70%. As a proof of concept, we integrate a quantum photonic circuit with six independently operated and deterministically integrated quantum emitters on the same photonic chip. An artistic image of proposed architecture is shown in Fig. 5j while the inset shows a microscope image of the fabricated device. The nanowire quantum emitters are encapsulated in SiN waveguides and pumped with an in-plane HeNe laser. The different colours of emissions in the waveguides present photons generated in different QDs. The emission spectra of the quantum emitters are shown in Fig. 5d–i, colour coded to the artistic image. The measured QD spectra depends on the initial properties of the selected wires, with no limitation the selection criteria during transfer. Our fast deterministic integration scheme (more details in Supplementary Note 7) along with the possibility to perform three-dimensional (3D) integration with SiN, allows for realizing more complex architectures with large range of wavelength operation.

## Discussion

In summary, we have deterministically integrated quantum emitters on a complementary metal–oxide–semiconductor (CMOS) compatible platform with tunable ring resonator filters that show ultra-efficient pump rejection. This allowed us to demonstrate a reconfigurable 2-emitter WDM channel, which can be extended to include more quantum emitters and incorporate additional coding schemes. By eliminating the need for off-chip components and providing in-plane excitation scheme, our results open up new possibilities for large-scale quantum photonic systems with on-chip single- and entangled-photon sources. Our approach, providing efficient tunable filtering and routing of single photons, allows for the integration of complex architectures and will enable experiments far beyond two particles. We believe that the presented approach using nanowires, which offer high degree of growth control, is the most promising integration technique. Especially, since crystal phase QDs in nanowires are emerging as a highly designable system at the atomic layer level^35, 36^. This brings us an important step closer to realize the ambitious schemes of (linear) optical quantum computing that have been put forward over the past decades.

## Methods

### Nanomanipulator

This tool is capable of positioning and aligning nano-sized objects with < 250 nm position resolution and <1° rotation resolution. It consists of a tungsten tip mounted on a *xyz* high-precision differential stage, all integrated in a high-resolution imaging system. Relying on van der Waals forces between the nanowires and the tungsten tip, nanowires can be selectively transferred from the growth chip to another substrate for further processing. More details about the nanowire transfer process and the nanomanipulator can be found in ref. ^7^.

### Photonic circuit fabrication: encapsulation device

Starting with a bare silicon wafer, 2.4 µm of thermal oxide is formed to serve as the bottom cladding of the waveguide. Using e-beam lithography, metal evaporation, and lift-off process, metallic structures, including marker fields and heaters (resistance 2.8 kΩ) were created on the oxide layer. Next, nanowire QDs were transferred with the nanomanipulation tool to the substrate, followed by a deposition of 200 nm of SiN using plasma-enhanced chemical vapour deposition (PECVD) process at 300 °C^28^. Waveguides and ring resonators were patterned using 100 keV e-beam lithography on a 950 K PMMA resist. After developing the resist, features were transferred to the SiN by complete etching of the SiN layer using CHF3/Ar-based reactive ion etching. This was followed by a short O2 plasma-cleaning step. Next, the devices were covered with ~ 1 μm-thick PMMA to provide symmetric mode confinement and negative thermo-optic tuning of the rings. Finally, to reduce the thermal coupling between the heaters and the nanowires, and to provide easy access to the electrical contacts for bonding, the PMMA layer was removed from the majority of the substrate, except in regions surrounding the photonic circuit. The authors used SiN as the photonic channel core due to a number of attractive properties. It has one of the largest energy bandgaps (5 eV), allowing for a wide range of operating wavelengths. Additionally, the material can be deposited allowing for complex 3D integration, which is bound to increase the photonic circuit density and expand the complexity of the realized architectures.

### Photonic circuit fabrication: butt coupling devices

This process is similar to the encapsulation devices for heaters (resistance 0.9 kΩ) and photonic element fabrication, except that nanowires are transferred after the circuit is fabricated. After cladding the whole chip with PMMA, an additional e-beam lithography step was performed to form openings in the cladding at the nanowire-waveguide coupler region. Finally, the nanowires are transferred and aligned using the nanomanipulation tool. The approach provides more flexibility in which the quantum sources are transferred after the photonic circuits are fabricated and tested, this enables more control and selectivity in matching the photonic circuits with the quantum emitters.

### QD excitation and photon collection

The chip design employed a U-shaped structure^28^ with input and output waveguide separation of 40 μm. This simplifies coupling to/from the side facet of the chip and separates input and output beams spatially with a two foci setup. The chip was cleaved along a crystallographic direction to achieve flat/smooth facet for coupling. Photons propagating in the SiN waveguides were collected using a ×50 objective with a numerical aperture of 0.75. The QDs can be either excited in-plane through the waveguide using a HeNe laser (632 nm), or out-of-plane using a solid-state green laser (532 nm).

### Data availability

The data supporting the findings of this study are available from the corresponding author on reasonable request.

## Electronic supplementary material

Supplementary Information
