# Supplementary material for: On-chip single photon filtering and multiplexing in hybrid quantum photonic circuits
Source: Nat Commun. 2017 Aug 30;8:379. doi: 10.1038/s41467-017-00486-8 (PMC5577278; doi:10.1038/s41467-017-00486-8)
Supplement: Supplementary file 1 — Supplementary Information [file 41467_2017_486_MOESM1_ESM.pdf]

Description of Supplementary Files

File Name: Supplementary Information

Description: Supplementary Figures, Supplementary Notes, and Supplementary References

Supplementary Notes

Supplementary Note 1

As discussed in the main manuscript, the waveguide dimensions (800 nm 200 nm) and gap between the ring-waveguide and the bus-waveguide (180 nm) are all optimized to achieved critical coupling for TE resonant modes. The TM resonant modes are critically coupled at larger waveguide separations (~340 nm), while being over-coupled in the presented devices with 180 nm gaps ($Q_{\text{coupling}} < Q_{\text{intrinsic}}$). The through-port transmission of the TM resonant modes is shown in **Supplementary Figure 1**, the resonances show lower loaded quality factor than the TE modes due to the additional loss rate provided by the leakage to the bus-waveguides.

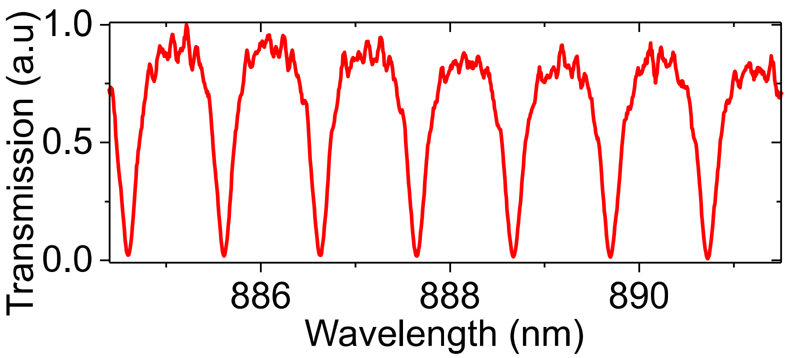

Supplementary Figure 1 |TM mode through-port transmission in the ring resonator. The ring resonator was designed to be critically coupled for the TE mode near the QD emission wavelength. The figure shows the transmission of the TM mode, where resonator is operating in the over-coupled regime.

Supplementary Note 2

In order to accurately determine the QD temperature while tuning the ring resonator filter, we studied the QD emission wavelength shift as a function of the cryostat temperature. As shown in **Supplementary Figure 2**, the emission wavelength is a non-linear function of the temperature. By comparing the QD emission wavelength shift at a specific voltage with the wavelength shifts at a certain cryostat temperature, we can deduce the QD temperature while tuning the ring resonator filter. We estimated the QD temperature at 12.5 Volts of tuning voltage to be < 35 K.

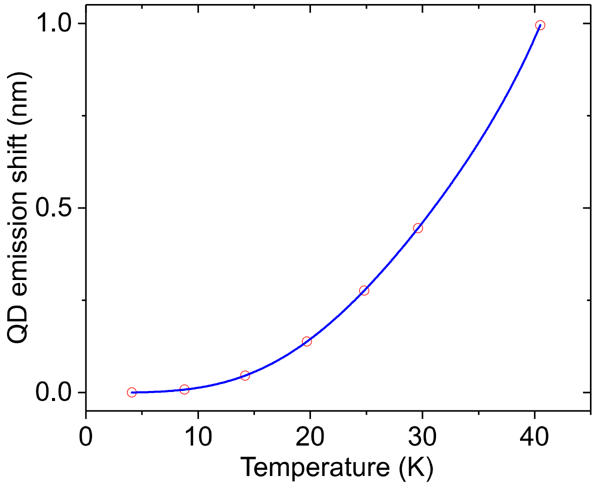

Supplementary Figure 2 | Quantum dot emission wavelength tuning. To couple the quantum dot emission to the ring resonator, the exciton wavelength and the ring resonance must be matched. The figure shows the QD emission wavelength shift as a function of the substrate temperature, where the red circles present the measured data and the blue line presents the data fit.

Supplementary Note 3

The excellent on-chip filtering that is demonstrated can be attributed to a combination of attenuation and filtering of unwanted photons. Ultra-efficient pump-suppression in the drop port is a result of material absorption, severe under-coupling of the pump to the resonator, and the use of top excitation. The high-frequency chemical vapor deposition of SiN (1:1 NH_3:SiH_4) exhibits high optical absorption at the visible range of the spectrum[1]. Additionally, the pump photons have much lower coupling to the resonating modes of the cavity. The ring-waveguide gap of 180 nm was designed to achieve critical coupling of TE modes at a wavelength of ~880 nm. For the pump photons, the effective length of the two coupling gaps is increased by ~400 nm, resulting in close to zero transmission to the drop port. A similar argument is valid for the bulk InP nanowire emission at approximately 830 nm [2] shown in **Supplementary Figure 3** with excitation power $P_{\text{excitation}} >> P_{\text{saturation}}$. Therefore, the bulk InP emission couples less efficiently to the drop port.

Furthermore, since the waveguide dimensions impose a single mode cut-off for pump photons, the pump is coupled to higher order modes which exhibit weak coupling to the resonant modes of the cavity due to the small overlap integral between the evanescent fields.

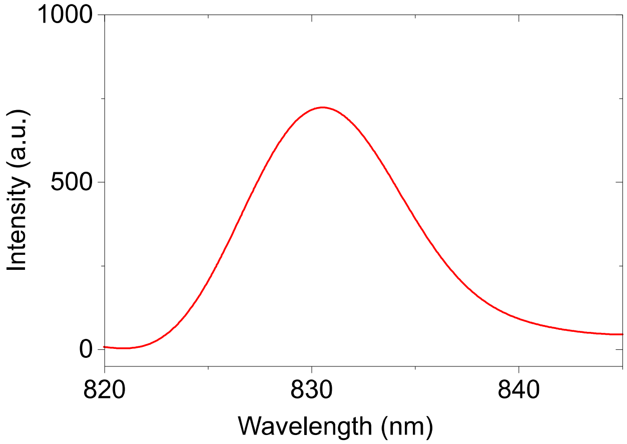

Supplementary Figure 3 | Nanowire bulk InP emission. The filtering of bulk nanowire emission is crucial for realizing quantum light on-chip. The figure shows typical bulk emission centered at ~ 830 nm, which is approximately 50 nm less than the emission wavelength of the quantum dots used in the study.

Supplementary Note 4

The slow detector response limits the resolution of the measured correlation functions. We independently measured the response time of the Avalanch Photodiode (APD) using a 3 picoseconds pulsed laser to be >350 ps, shown in **Supplementary Figure 4**. We have omitted the use of superconducting single photon detectors with better time resolution in the correlation measurements to avoid off-chip spatial filtering through coupling to single mode optical fibers. The slow detector response was included in the measurement through convoluting the setup response with an ideal second-order correlation measurement in conjunction with multi-dimensional

optimization (the decay rate, the depth of the 0-delay point, and the position of the 0-delay point) to minimize the fitting error to the measured date.

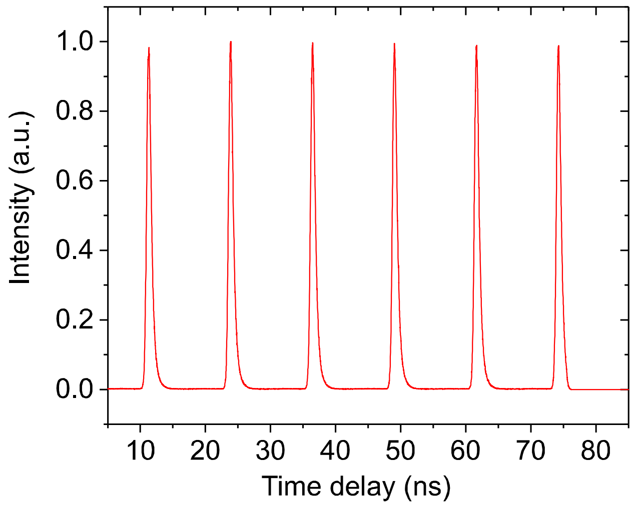

Supplementary Figure 4 | Time response of the setup. Measured setup response for a 3 ps pulsed laser.

By incorporating the response function of the detectors, we directly fit the measured the second-order correlation functions limited by the detector response (green line in **Figure.3d** and **Figure.3e** in the manuscript). The data fit yields $g^2(0) = 0.59 \pm 0.05$ for the ring resonator filtering and $g^2(0) = 0.39 \pm 0.04$ for the monochromator filtering.

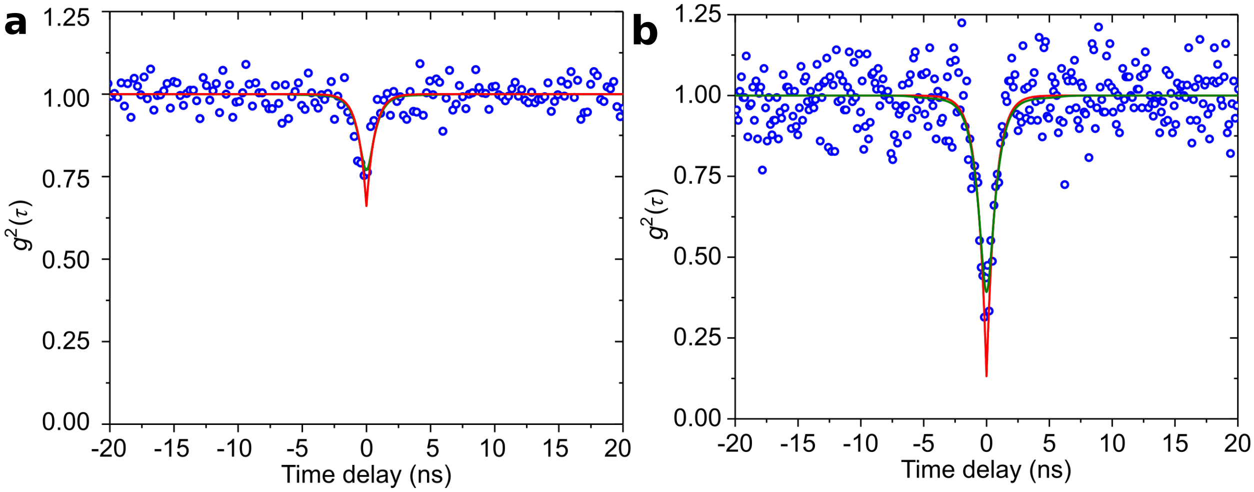

Supplementary Figure 5 | Correlation measurements. (a) Auto-correlation measurement with ring resonator filtering considering both TE and TM modes at the same time, with QD emission aligned to TE resonance of the ring resonator. (b) cross-correlation measurement between the trion and exciton emission lines in **Figure.3b** in the manuscript. The blue circles present the measured data while the red and green lines are data fits omitting and considering detector response time, respectively.

Supplementary Figure 5a shows the second-order correlation function measured with only the ring resonator filtering, similar to **Figure.3d** in the main manuscript, but we consider both TE and TM modes at the same time. The multi-photon probability at zero time delay was measured to be $g^2(0)$ = 0.66 ± 0.03, considering the detector response. The value is slightly larger than the TE value presented in the main manuscript due to the finite contribution of the TM bulk InP nanowire emission through the over-coupled TM resonant modes. **Supplementary Figure 5b** shows cross-correlation measurement on the trion and exciton lines of the nanowire shown in

Figure.3b in the main manuscript. The multi-photon probability at zero time delay was measured to be $g^2(0) = 0.13 \pm 0.06$ ($g^2(0) = 0.23 \pm 0.06$ without considering the detector response).

Finally, previously measured values of encapsulated nanowires, ref [11] in the main manuscript, show multiphoton probability at zero delay equal to 0.07 using spectrometer filtering without any detector correction. Photonic circuit integration has no effect on the quality of the photon emission. Therefore, the presented measurements in the paper provide a proof-of-principle experiment in a new territory of selective integration and filtering of quantum emitters, and by no means the reported $g^2(0)$ values provide the limit on what can be done.

Supplementary Note 5

We performed finite difference time domain simulations to estimate the coupling efficiency between the butt-coupled nanowires and the photonic waveguide. The simulated structure and a horizontal cut depicting the electric field distribution of the TE mode are shown in **Supplementary Figure 6a** and **Supplementary Figure 6b**, respectively**.** The coupling efficiency to the waveguide was calculated to be 3% of the total forward emitted power in the nanowire. The resonators in the multiplexing circuits are designed to have smaller gaps to increase coupling to the drop port. Nanowire encapsulation and the use of 1D Bragg reflectors can be used to

achieve a uni-directional emission with much higher efficiencies exceeding 90% [3].

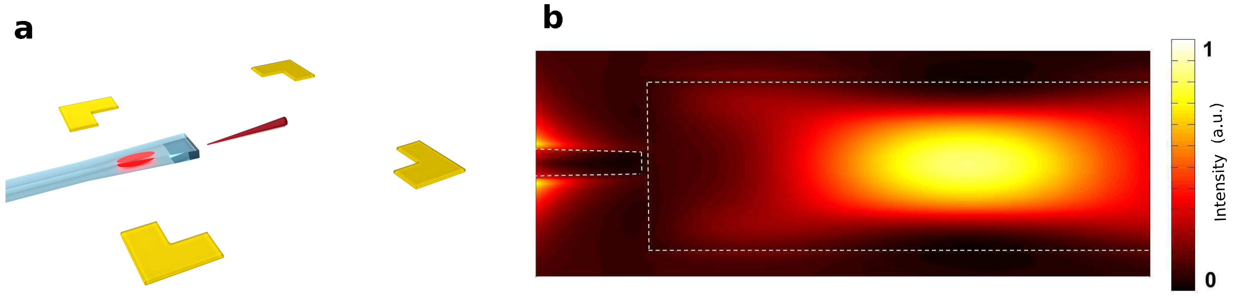

Supplementary Figure 6 | Nanowire waveguide butt coupling. (a) Simulated device of a nanowire QD butt coupled to a photonic waveguide.(b) Electric field distribution of the TE mode emitted from nanowire QD to the photonic waveguide. In (b) the dashed while lines define the outline of the SiN waveguide (right) and the nanowire tip (left).

Supplementary Note 6

Here we present the results of another WDM device. Two nanowires QDs are butt coupled to a SiN waveguide, their emission wavelengths are separated by ~6 nm. As shown in **Supplementary Figure 7,** we are able to wavelength-multiplex the emission of both QDs into one photonic channel, then demultiplexing the emission using a tunable filter. As we increase the tuning voltage, QD1 can be decoupled from the through-port of the ring resonator leaving only the signal from QD2. In this device, the voltages

applied are higher than the QWDM device presented in the main manuscript, this is attributed to the fact that the we used an off-chip heater for tuning.

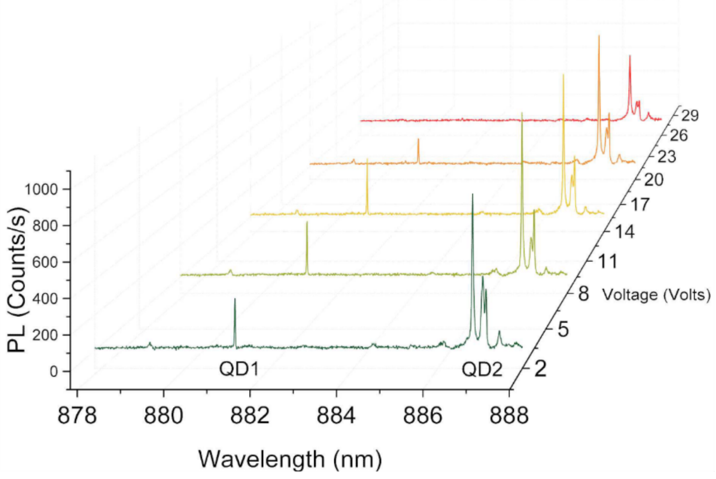

Supplementary Figure 7 | Wavelength division multiplexing of nanowire quantum dots. Through-port transmission of the multiplexed photonic channel as a function of the heater voltage voltage. At low voltages, both QDs are wavelength multiplexed into a single photonic channel. As the voltage is increase, QD1 can be filtered-out from the through-port of the ring resonator leaving only the signal from QD2.

Supplementary Note 7

Our current technology allows for transfer of wires on an average time of 6 min with >70% success rate. As a proof of concept, we integrated more complex quantum photonic circuits with selected 6 sources operating independently with no significant additional effort. **Supplementary Figure 8** shows the transfer process of site selected nanowire quantum dots from the growth chip. **Supplementary Figure 9a** shows the wires transferred to

the circuit chip. **Supplementary Figure 9b** and **Supplementary Figure 9c** shows the photonic circuit after etching, while **Supplementary Figure 9d** shows close-up image of the tunable ring resonator filter. The emission spectra of the nanowires are included in **Figure.5** of the main manuscript. All the wires are pumped in-plane using a HeNe laser. The presented excitation scheme paves the way for large-scale integration where a single source, can be electrically-pumped[4], is used to excite multiple quantum emitters using integrated routers and filters. In order achieve scalable integration of larger number of sources, an automated process needs to be implemented. More interestingly with the current approach, our NWs are all site selected so in principle a fully-automated process can be performed to pick NW from the growth chip, then transfer it to the pre-marked place on the target chip. Finally, finding two or more identical quantum dots to perform quantum interference measurements is challenging. But, we believe that our approach using nanowires, which offer a higher degree of growth control, is one of the most promising quantum photonic integration techniques. Especially since crystal phase quantum dots are emerging as a highly designable system at the atomic layer level[5].

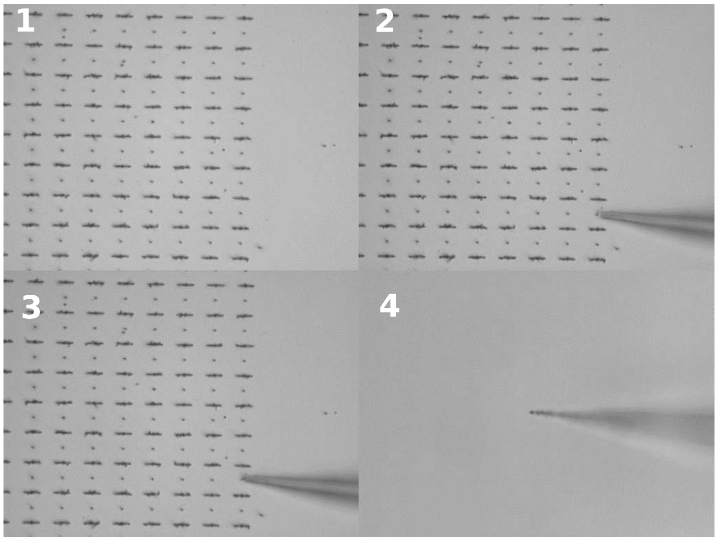

Supplementary Figure 8 | Nanowire transfer process. Deterministic transfer of selective nanowires. (1) Position-selected nanowires shown in the growth chip. (2) Nanomanipulator tip approaches to transfer the nanowires. (3) The nanowire is detached from the growth chip and transferred (4) to the photonic circuit chip.

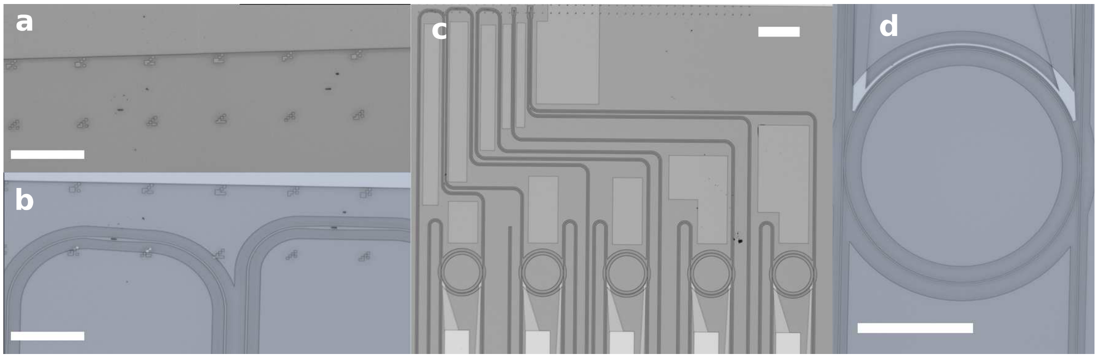

Supplementary Figure 9 | Fabrication of six quantum emitters in a single photonic circuit. Integrated complex photonic circuit with 6 independently operating sources. (a) nanowires transferred to the photonic circuit then encapsulated with SiN. (b) Photonic circuit and waveguides are

etched. (c) image of the 6-quantum emitter photonic circuit. (d) close-up of the ring resonator filter. The scale bars of (a), (b), (c) and (d) are 30 μm, 30 μm, 140 μm, and 70 μm respectively.

Supplementary references :

[1] A. Gorin, A. Jaouad, E. Grondin, V. Aimez, and P. Charette, "Fabrication of silicon nitride waveguides for visible-light using PECVD: a study of the effect of plasma frequency on optical properties," Optics Express, 16, 13509-13516 (2008).

[2] K. D. Jöns, L. Schweickert, M. A. M. Versteegh, D. Dalacu, P. J. Poole, A. Gulinatti, et al., "Bright nanoscale source of deterministic entangled photon pairs violating Bell’s inequality," Scientific Reports, **7**, 1700 (2017).

[3] I.E. Zadeh, A.W. Elshaari, K.D. Jöns, A. Fognini, D. Dalacu, P.J. Poole, M.E. Reimer, V. Zwiller, “Deterministic Integration of Single Photon Sources in Silicon Based Photonic Circuits” Nano Letters, 16, 2289-2294 (2016).

[4] J.P. Lee, E. Murray, A.J. Bennett, D.J.P. Ellis, C. Dangel, I. Farrer, P. Spencer, D.A. Ritchie, A.J. Shields,”Electrically driven and electrically tunable quantum light sources”, Applied Physics Letters, 110, 071102 (2017).

[5] N. Akopian, G. Patriarche, L. Liu, J.C. Harmand, V. Zwiller,”Crystal Phase Quantum Dots”, Nano Letters, 10, 1198-1201 (2010).
